# Constructing critical thinking in health professional education

**DOI:** 10.1007/s40037-018-0415-z

**Published:** 2018-04-04

**Authors:** Renate Kahlke, Kevin Eva

**Affiliations:** Centre for Health Education Scholarship, University of British Columbia, Vancouver, Canada

**Keywords:** Critical thinking, Clinical reasoning, Health professions education

## Abstract

**Introduction:**

Calls for enabling ‘critical thinking’ are ubiquitous in health professional education. However, there is little agreement in the literature or in practice as to what this term means and efforts to generate a universal definition have found limited traction. Moreover, the variability observed might suggest that multiplicity has value that the quest for universal definitions has failed to capture. In this study, we sought to map the multiple conceptions of critical thinking in circulation in health professional education to understand the relationships and tensions between them.

**Methods:**

We used an inductive, qualitative approach to explore conceptions of critical thinking with educators from four health professions: medicine, nursing, pharmacy, and social work. Four participants from each profession participated in two individual in-depth semi-structured interviews, the latter of which induced reflection on a visual depiction of results generated from the first set of interviews.

**Results:**

Three main conceptions of critical thinking were identified: biomedical, humanist, and social justice-oriented critical thinking. ‘Biomedical critical thinking’ was the dominant conception. While each conception had distinct features, the particular conceptions of critical thinking espoused by individual participants were not stable within or between interviews.

**Discussion:**

Multiple conceptions of critical thinking likely offer educators the ability to express diverse beliefs about what ‘good thinking’ means in variable contexts. The findings suggest that any single definition of critical thinking in the health professions will be inherently contentious and, we argue, should be. Such debates, when made visible to educators and trainees, can be highly productive.

**Electronic supplementary material:**

The online version of this article (10.1007/s40037-018-0415-z) contains supplementary material, which is available to authorized users.

## What this paper adds

‘Critical thinking’ is a term commonly used across health professional education, though there is little agreement on what this means in the literature or in practice. We depart from previous work, which most often attempts to create a common definition. Instead, we offer a description of the different conceptions of critical thinking held in health professional education, illustrate their dynamic use, and discuss the tensions and affordances that this diversity brings to the field. We argue that diversity in conceptions of critical thinking can allow educators to express unique and often divergent beliefs about what ‘good thinking’ means in their contexts.

## Introduction

Even though the term *critical thinking* is ubiquitous in educational settings, there is significant disagreement about what it means to ‘think critically’ [1]. Predominantly, authors have attempted to develop consensus definitions of critical thinking that would finally put these disagreements to rest (e. g. [2–5]). They define critical thinking variously, but tend to focus on a rational process involving (for example) ‘interpretation, analysis, evaluation, inference, explanation, and self-regulation’ [2]. Other authors have challenged this perspective by arguing that critical thinking is a more subjective process, emphasizing the role of emotion and relationships [6–9]. In the tradition of critical pedagogy, critical thinking has meant critiquing ideology [10–12]. Last, still others have argued that critical thinking is discipline or subject-specific, meaning that critical thinking is not universal, but does have a relatively stable meaning within different disciplines [13–18]. However, none of these attempts to clarify the ambiguity that surrounds *critical thinking *have led to agreement, suggesting that each of these perspectives offers, at best, a partial explanation for the persistence of disagreements.

This is problematic in health professional education (HPE) because professional programs are mandated to educate practitioners who have a defined knowledge base and skill set. When curriculum designers, educators, researchers, or policy-makers all agree that we should teach future professionals to ‘think critically’, resting on the assumption that they also agree on what that means, they may find themselves working at cross-purposes. Moreover, the focus on a stable meaning for critical thinking, whether within a discipline or across disciplines, cannot account for the potential value of the multiplicity of definitions that exist. That is, the availability of diverse conceptions of critical thinking likely enables educators to express diverse elements of and beliefs about their work, thereby suggesting a need to explore the conceptions of critical thinking held in HPE, and the contexts that inform those conceptions.

With the historical focus on developing broad definitions of critical thinking and delineating its component skills and dispositions, little has been done either to document the diverse conceptions of this term in circulation amongst active HPE practitioners or, perhaps more importantly, to illuminate the beliefs about what constitutes ‘good thinking’ that lie behind them and the relationships between them. Perhaps clarity in our understanding of critical thinking lies in the flexibility with which it is conceptualized. This study moves away from attempting to create universal definitions of critical thinking in order to explore the tensions that surround different, converging, and competing beliefs about what critical thinking means.

In doing so, we map out conceptions of critical thinking across four health professions along with the beliefs about professional practice that underpin those conceptions. Some of these beliefs may be tied to a profession’s socialization processes and many will be tied to beliefs about ‘good thinking’ that are shared across professions, since health professionals work within shared systems [19] toward the same ultimate task of providing patient care. It is the variety of ways in which critical thinking is considered by practitioners on the whole that we wanted to understand, not the formal pronouncements of what might be listed as competencies or components of critical thinking within any one profession.

Hence, with this study, we sought to ask:

1. How do educators in the health professions understand critical thinking?
2. What values or beliefs inform that understanding?

To explore these questions, we adopted a qualitative research approach that focuses on how people interpret and make meaning out of their experiences and actively construct their social worlds [20].

## Methods

This study uses an emergent, inductive design in an effort to be responsive to the co-construction of new and unexpected meaning between participants and researchers. While techniques derived from constructivist grounded theory [21] were employed, methods like extensive theoretical sampling (that are common to that methodology) were not maintained because this study was intended to be broadly exploratory. This ‘borrowing’ of techniques offers the ability to capitalize on the open and broad approach offered by interpretive qualitative methodology [20] while engaging selectively with the more specific tools and techniques available from constructivist grounded theory [22, 23].

The first author has a background in sociocultural and critical theory. Data collection and early analyses were carried out as part of her dissertation in Educational Policy Studies. As a result of her background in critical theory, there was a need for reflexivity focused on limiting predisposition toward participant interpretations of critical thinking that aligned with critical theory. The senior author was trained in cognitive psychology, and contributed to the questioning of results and discussion required to ensure this reflexivity. The first author’s dissertation supervisor also provided support in this way by questioning assumptions made during the initial stages of this work.

### Sampling

Participants were recruited through faculty or departmental listservs for educators. Senior administrators were consulted to ensure that they were aware of and comfortable with this research taking place in their unit. In some cases, administrators identified a few key individuals who were particularly interested in education. These educators were contacted directly by the first author to request participation.

The purposive sample includes four educators from each of four diverse health professional programs (*n* = 16 in total): medicine, nursing, pharmacy, and social work. All participants self-identified as being actively involved in teaching in their professional program and all were formally affiliated with either the University of Alberta (Medicine, Nursing, and Pharmacy) or the University of Calgary (Social Work). These four professions were selected to maximize diversity in approaches to critical thinking given that these professions have diverse perspectives and roles with respect to patient care. However, participants all worked in Alberta, Canada, within the same broad postsecondary education and healthcare contexts.

In addition, sampling priority was given to recruiting participants practising in a diverse range of specialties: primary care, geriatrics, paediatrics, mental health, critical care, and various consulting specialties. Specific specialties within each profession are not provided here in an effort to preserve participant anonymity. The goal was not to make conclusions about the perspective of any one group; rather, diversity in profession, practice context, gender, and years in practice was sought to increase the likelihood of illuminating diverse conceptions of critical thinking.

### Data generation

Participants were invited to participate in two in-person semi-structured interviews conducted by the first author. All but one participant completed both interviews. Interviews were audio-recorded and interview guides are included in the online Electronic Supplementary Material. The first was about 1 hour in length and discussed how participants think about critical thinking in their teaching, professional practice, and other contexts. Participants were invited to bring a teaching artefact that represented how they teach critical thinking to the interview. Artefacts were used as a visual elicitation strategy to prompt discussion from a new angle [24]. Questions focused on what the participant thought about teaching critical thinking using the artefact and how they identified critical thinking (or lack thereof) in their students. Artefacts were not analyzed independently of the discussion they produced [25].

Interview 1 data were analyzed to produce a visual depiction of the aggregate terms, ideas, and relationships described by participants. The visual depiction took the form of a ‘mind map’ (see Appendix C of the Electronic Supplementary Material) that was generated using MindMup free online software [26]. In developing the mind map, we sought descriptions of participants’ views that remained as close to the data as possible, limiting interpretations and inferences. The ‘clusters’ that appear in the mind map (e. g., the cluster around ‘characteristics of the critical thinker’) represent relationships or categories commonly described when participants discussed those terms. Terms were not weighted or emphasized based on frequency of use (through font size or bolding) in an effort to allow individual participants to emphasize or deemphasize terms as they thought appropriate during the second interview.

Where there was no clear category or relationship, terms were left at the first level of the mind map, connected directly to ‘critical thinking’ at the centre. Including more connections and inferences would likely have improved the readability of the map for participants; however, we chose to include connections and exact language used by participants (even in cases where terms seemed similar) as often as possible, in an effort to limit researcher interpretation. That said, any attempt to aggregate data or to represent relationships is an act of interpretation and some inferences were made in the process, such as the distinction between descriptions about ‘characteristics’ of the critical thinker (the top left hand corner of the map) and ‘processes’ such as ‘reasoning’ or ‘examining assumptions’ (on the right side of the map). The second interview lasted approximately 45 minutes during which a visual elicitation approach invited participants to respond to the mind map.

Visual elicitation involves employing visual stimuli to generate verbal interview data. Participant-generated mind maps are often used in qualitative data collection [27], but the literature on using researcher-generated diagrams for visual elicitation is relatively thin [25, 28]. In this study, using a researcher-generated mind map for visual elicitation offered several advantages. First, as with other forms of visual elicitation, diagrams of this kind can help participants develop candid responses and avoid rehearsed narratives [24, 29]. For example, we used mind maps as one mechanism to reduce the tendency for participants who were familiar with the literature on critical thinking to get stuck on narrating seemingly rehearsed definitions of critical thinking. Second, we chose to use a mind map because it provided a social setting through which participants could react to language generated by others. Doing so does not allow the same degree of social negotiation inherent in focus groups, but it avoids the difficulty involved in attempts to disentangle individual from group views [30]. Third, the visual elicitation method was chosen because it offered a form of member check [31] that allowed researchers to understand the evolving nature of participants’ conceptions of critical thinking, rather than assuming that participants offer a single true conception during each and every discussion [32]. In other words, the mind map was used to prompt participants to elaborate their conception of critical thinking and locate it relative to other participants.

In interview 2, participants were asked to begin by discussing areas or terms on the mind map that resonated most with their own conception of critical thinking; they were then asked to discuss terms or concepts on the map that resonated less or with which they disagreed. They were also asked to comment on how relationships between ideas were represented through the map so that researchers could get a sense of the extent to which the relationships between the concepts depicted reflected the participants’ understanding of those relationships [28]. Participants were encouraged to disagree with portions of the map and most did actively disagree with some of the terms and relationships depicted, suggesting that the map did not come to dictate more than elicit individual interpretations [28]. Although participants were encouraged to ‘mark up’ the mind map, and the ‘marked up’ mind maps were treated as data, the primary data sources for this study were the audio-recorded interviews [25].

Participants were aware that the mind map represented aggregate data from the four health professions in the study, but were not initially told whether any of the responses came predominantly from any one profession; they did not generally seem to be attempting to associate terms with other professions. Nonetheless, interview 2 data are a mix of participants’ reactions to the ideas of others and their elaborations of their own understandings. Naturally, these data build on data generated in interview 1, and represent reactions to both the researcher interpretation of the data and to the conceptions of critical thinking offered by others. Interview 1 data tended to offer an initial, open impression of how participants think about critical thinking in their contexts. Because of these different approaches to data generation, quotes from interview 1 and 2 are labelled as ‘INT1’ or ‘INT2’, respectively.

### Data analysis

Data were coded through an iterative cycle of initial and focused coding [33] with NVivo software. Initial line-by-line coding was used to develop codes that were close to the data, involving minimal abstraction. Initial codes were reviewed by the first author and dissertation supervisor to abstract categories (conceptions of critical thinking), sub-categories (features of those conceptions), and themes related to the relationships between those categories. Focused coding involved taking these categories and testing them against the data using constant comparison techniques derived from constructivist grounded theory [21]. Category development continued during the framing of this paper, and authors engaged in ongoing conversations to modify categories to better fit the data. In this process, we returned to the data to look for exceptions that did not fit any category, as well as contradictions and overlap between categories.

Interpretive sufficiency [34], in this study, occurred when no new features illustrating participants’ conceptions of critical thinking were identified. Memos were kept to track the development or elimination of initial insights or impressions. Institutional ethics approval was obtained from the University of Alberta.

Participant identities have been masked to preserve anonymity. The abbreviation ‘MD’ refers to educators in medical education, ‘NURS’ to nursing, ‘PHARM’ to pharmacy, and ‘SW’ to social work. Participants within each group were then assigned a number. For example, the code NURS3 is a unique identifier for a single participant.

## Results

Three main conceptions of critical thinking were identified, each of which will be elaborated in greater detail below: biomedical critical thinking, humanist critical thinking, and social justice-oriented critical thinking. It is important to note that these categories focus on the process and purpose of critical thinking, as defined by participants. Participant comments also spoke to the ‘characteristics’ or ‘dispositions’ of critical thinkers, such as ‘open-mindedness’ or ‘creativity’. The focus of this study, however, was on uncovering what critical thinking looks like as opposed to what a ‘critical thinker’ looks like.

The results below interweave responses from different professional groups in order to emphasize the way in which each of the three core conceptions that we have identified crosses professional boundaries. We then provide a brief discussion of the relationships between these three conceptions, emphasizing the limited extent to which these conceptions were profession-specific, and the tensions that we observed between these conceptions. In general, we also interweave results from both interviews because the discussion in interview 2 tended to reinforce the themes arising from interview 1, especially with respect to indications that different conceptions were used fluidly by individuals over time and dependent on the context being discussed. The interview from which data arose is marked after each quote and we have mentioned explicitly whenever a comment was made in specific response to the mind map presented during interview 2.

In this way, our data extend the literature on critical thinking by offering an appreciation of how each of these conceptions provide educators a different way of thinking, talking, and teaching about their work in HPE. We found that even individual participants’ conceptions of critical thinking shifted from time to time. That is, they often articulated more than one understanding of critical thinking over the course of an interview or between interviews 1 and 2. Some of these conceptions were shared by multiple participants but individual constellations of beliefs about what critical thinking means were unique and somewhat idiosyncratic. Thus, while participants’ conceptions of critical thinking were both idiosyncratic and common, they were also flexible and contextual; the meaning of critical thinking was continuously reconstructed and contested. In this way, *critical thinking *offered a window through which to explore how beliefs about what constitutes ‘good thinking’ in a profession are challenged in educational settings.

### Biomedical critical thinking

Participants articulating a biomedical approach saw critical thinking and clinical reasoning as nearly synonymous. They emphasized a process that was rational, logical, and systematic. One participant articulated that critical thinking is ‘*to be able to reason logically’* (NURS4 INT1). Another related:

*You have to kind of pull together data that’s relevant to the subject you’re dealing with. You have to interpret it, you have to analyse it, and you have to come up with some type of conclusions at the end as to how you deal with it.* (PHARM3 INT1)

Participants discussing this approach agreed that critical thinking involved a systematic process of gathering and analyzing data: *‘I think [critical thinking and clinical reasoning] are the same. I think clinical reasoning is basically taking the data you have on a patient and interpreting it, and offering a treatment plan’ *(MD1 INT1).

In keeping with an emphasis on the rational and logical, participants espousing this view often reacted negatively when they saw references to emotion on the mind map in interview 2: *‘as soon as you bring your emotions into the room, you’re no longer applying what I think is critical thinking’* (MD4 INT2). Participants also noted that decision-making was an important component of critical thinking: ‘*you have to make a decision. I think it’s a really important part of it’* (MD2 INT2).

For participants from pharmacy, in particular, critical thinking often meant departing from ‘rules’ that guide clinical practice in order to engage in reasoning and make situationally nuanced decisions. One pharmacist, describing a student not engaging in critical thinking, related that the student asked:

*‘Have you ever seen Victoza given at 2.4* *milligrams daily?’ … It’s very, you know, it’s very much yes or no. But at a deeper level, it’s actually missing things. … [There are] all these other factors that change the decision, right? … On paper there might be a regular set of values for the dose, … [but] without the rest of the background, that’s a very secondary thing. *(PHARM4 INT1)

This perspective was identified as the dominant conception of critical thinking because the terms and concepts falling under this broad approach were most frequently discussed by participants; moreover, when participants discussed other conceptions of critical thinking, they were often explicitly drawing contrast to the biomedical view. While the biomedical perspective was dominant in all four groups (although primarily as a contrasting case for social workers), participants tended to occupy more than one perspective over the course of an interview. They might talk primarily about biomedical critical thinking, but also explicitly modify that perspective by drawing on the other two approaches identified: humanist critical and social justice-oriented critical thinking.

### Humanist critical thinking

Participants, when adopting this view, described critical thinking as directed toward social good and oriented around positive human relationships. Humanist conceptions of critical thinking were often positioned as an alternative to the dominant biomedical perspective: *‘having to think of somebody else, at their most vulnerable, makes you know that knowledge alone, science alone, won’t get that patient to the place you want the patient to be. It won’t provide the best care’* (NURS1 INT1). In being so positioned, the humanist conception of critical thinking explicitly departed from the biomedical, which emphasized ‘setting aside’ emotion and de-emphasized the role of relationships in healthcare. In the humanist perspective, participants often discussed the purpose of critical thinking as:

*Thinking about something for the betterment of yourself and the betterment of others. We’re social beings as human beings. … I think [critical thinking] has a higher purpose. … But I think that [if] critical thinking … [is] a human trait that we have or hope to have, then it has to have those components of what we are as humans.* (NURS1 INT1)

Another participant emphasized that: *‘a great part of critical thinking is that human element and the consideration of ultimately what’s a good thing, a common good’* (NURS2 INT1).

In addressing the relational aspects of humanist critical thinking, participants argued that the focus on ‘hard’ sources of data, such as lab tests or imaging, in biomedical critical thinking was limiting. They were concerned that ‘hard data’ tend to be perceived as more objective and thus more important in biomedical critical thinking, compared with subjective patient narratives. They argued that the patient’s story is essential to critical thinking:

*I think it doesn’t matter what kind of expert you are, you have to be able to think about patients in the context that they’re in and consider what the patient has to say, and really hear them. So I think that’s an important—that was a total lack of critical thinking in a totally, ‘I’m just going to get through this next patient to the next one’*. (MD1 INT1)

Taken together, these perspectives suggest that biomedical approaches to critical thinking fail to address the complex relational and psychosocial aspects of professional practice.

### Social justice-oriented critical thinking

In social justice-oriented approaches to critical thinking participants articulated a process of examining the assumptions and biases embedded in their world. They often explicitly rejected biomedical conceptions of critical thinking as ‘*reductionistic*’ (SW3 INT1) because, in their view, these approaches fail to address the thinker’s own biases. Educators taking a social justice approach felt that: *‘critical thinking … is around things like … recognizing your own bias and recognizing the bias in the world’* (SW1 INT1). In this perspective, participants saw critical thinking as a process of analyzing and addressing the ways in which individual and societal assumptions limit possible actions and access to resources for individuals and social groups.

Unlike biomedical critical thinking and similar to the humanist view, participants articulating this conception tended to make the values and goals of critical thinking, as they conceived of it, explicit. They often contrasted their articulation of values in critical thinking with the ‘assumed’ and unarticulated values present in the biomedical perspective:

*If you are not orientated in a social justice position, [critical thinking is] more about the mechanics, which is valuable as well, but … if we don’t understand the values associated with what we think, it seems to not be meaningless but there’s a piece missing or it’s assumed. The values are assumed.* (SW3 INT1)

When taking this perspective, participants argued that it is necessary to understand social systems in order to think critically about individual patient cases. One educator questioned:

*Why are there a disproportionate number of aboriginal inpatients than any other group? … When you start critically thinking about seeing the whole patient … there are issues related with all of society and that’s why people have more diabetes.* (PHARM1 INT1)

Other participants had measured responses to this approach. One participant added to their primarily biomedical approach in order to accommodate perspectives encountered in the mind map, relating that behind their diagnostic work all physicians:

*Certainly see a wide spectrum of social and economic status and cultures and things and recognizing that our system is kind of biased against certain groups as it is and knowing that but really not having a good sense of knowing even where to start deconstructing it. *(MD2 INT2)

### Relationships between conceptions of critical thinking

Results of this study suggest that critical thinking means a variety of things in different contexts and to different people. It might be tempting to see the three approaches outlined above as playing out along professional boundaries. Certainly, the social justice-oriented conception was more common among social work educators; the humanist approach was most common among participants from nursing; perspectives held by physician educators frequently aligned with dominant biomedical conceptions. In pharmacy, educators seemed to straddle all three perspectives, though they commonly emphasized a biomedical approach. Several participants suggested that their faculty or profession has a common understanding of critical thinking: ‘*critical thinking, for me and maybe for our faculty, is around things like …*’ (SW1 INT1).

However, while the disciplinary tendencies discussed above do appear in the data, these tendencies were not stable; participants often held more than one view on what critical thinking meant simultaneously, or shifted between perspectives. Participants also articulated approaches that were not common in their profession at certain moments, positioning themselves as ‘an outlier’, or positioning their specialty as having a different perspective than the profession as a whole, such that critical thinking might mean ‘thinking like a nurse’, or ‘thinking in geriatrics’. Further, participants’ perspectives shifted depending on the context in which they imagined critical thinking occurring.

This type of positioning and re-positioning occurred in both interviews, although they were particularly pronounced in interview 2, where participants were explicitly asked to react to different viewpoints by responding to the mind map. Examples of shifting perspectives in interview 1 occurred especially when participants from medicine shifted between biomedical and humanist conceptions. These shifts suggested a persistent tension and negotiation between characterizations of critical thinking as a rational process of data collection and analysis, and a more humanist approach that accounts for emotion and the relationship between professional and patient or family. Where participants sought to extend their notion of data beyond ‘hard data’ there is a sense of blending humanism with biomedical approaches to critical thinking. In the quote below, the participant brings together a call for a humanist relationship building with a need to gather and analyze all of the data, including important data about the patient’s experience:

*I have colleagues who’ll say [to their patients]: ‘just say yes or no.’ … And it’s not very good and they’re missing stuff. So, critical thinking is—I guess it’s sort of dynamic in that you have to have time and you also have to have an interaction.* (MD1 INT1)

While the participants described above negotiated between biomedical and humanist perspectives, participants primarily espousing a social justice-oriented conception of critical thinking responded to the ‘assumed’ values of the biomedical model. In talking about a problem solving-oriented biomedical approach, one participant argued that ‘*it’s important as well to have that, those foundational elements of how we think about what we think, but if we don’t understand the values associated … there’s a piece missing’* (SW3 INT1). Another stated that *‘critical thinking seems to be a neutral kind of process or—no, that can’t be true, can it?’* (SW1 INT2) with the mid-sentence shift indicating that two ways of conceptualizing critical thinking had come into conflict. This participant primarily discussed a social justice-oriented conception of critical thinking, which is not neutral, but at this moment also articulated a neutral, clinical reasoning-oriented or biomedical conception.

These relatively organic moments of negotiation certainly demonstrate a sense of conflicting values, of toggling between one perspective and another. However, they also suggest that there are ways in which these contradictions can be productively sustained. In negotiating between humanist and biomedical perspectives, educators effectively modify the dominant perspective.

In interview 2, when discussing the mind map, participants often encountered views that differed from their own. They responded either by making sense of and accommodating the new perspective, or by rejecting it. As an example of the former approach, one physician reacted to the ‘social justice-oriented’ corner of the mind map (specifically ‘examining assumptions’) by explaining how there are:

*Assumptions in the background that come up for me all the time in terms of the different ways people live and want to live and how we run into it all the time … it’s always in the background and actually influencing you and until someone challenges the way you approached something, you don’t know what your assumptions are.* (MD1 INT2)

As an example of a participant disagreeing with a perspective encountered in the mind map, one participant rejected social justice as an important component of critical thinking in medicine. They related that critical thinking has *‘got everything to do with reasoning, which makes sense. … Social justice has nothing to do with critical thinking’* (MD4 INT2). Interestingly, this participant also spoke at length about the link between social justice and critical thinking in the first interview, suggesting that a conception might seem ‘wrong’ when an individual is thinking and talking about it in one context, and entirely ‘right’ in another context.

Such results demonstrate that individual conceptions of critical thinking are multiple and flexible, not predetermined or stable. Educators bring certain values or perspectives into the foreground as they relate to the context under discussion, while others recede into the background. Though many participants seemed to have a primary perspective, multiple perspectives on critical thinking can co-exist and are actively negotiated by the individual.

## Discussion

In overview, the three broad conceptions of critical thinking offered here (biomedical, humanist, and social justice-oriented) echo approaches to critical thinking found in the critical thinking literature [11, 35–37]. However, this study extends the literature in two key ways. First, our data point to ways in which different conceptions of critical thinking conflict and coalesce, within the field, within each profession, and even within individuals. Second, this tension offers an early empirical account of critical thinking in the health professions that suggests there may be benefits to maintaining flexibility in how one conceives of the concept.

The diverse conceptions of critical thinking identified all appear to have some value in HPE. It might be tempting to view each conception as a unique but stable perspective, reflecting thinking skills that are used within a particular context or value orientation. However, the multiplicity and flexibility of participants’ conceptions in this study offers some explanation as to why previous attempts to develop either generic (e. g. [2, 3, 5]) or discipline-specific [13, 15–17] definitions and delineations of critical thinking have failed to stick.

Conceptions of critical thinking are not stable within a context or for a single educator. Educators’ conceptions of critical thinking shift within and between contexts as they navigate overlapping sets of values and beliefs. When educators take up different conceptions of critical thinking, the shifts they make are not just pragmatic; they actively negotiate the values and practices of the different communities in which they participate. Although we certainly saw hints of differences between professions, the strength of this study is that it captured the ways in which conceptions of critical thinking are not stably tied to any given profession. Critical thinking is connected to a broader idea of what ‘good thinking’—and, by extension, the ‘good professional’—looks like for each educator [38] within a given context or community.

These observations lead one to speculate about what purpose fluidity in conceptions of critical thinking might serve. Educators often have different values and goals for their profession, and, thus, it is not surprising that the meaning of critical thinking would be contested both within and across professions. Through their conceptions of critical thinking, participants contest ideas about what thinking is for in their profession—whether it should be focused on individual patient ‘problems’ or broader social issues, and the extent to which humanism is an important component of healthcare.

It is understandable that so much of the literature on critical thinking has sought to clarify a single ‘right’ definition; there is an argument for making a collective decision about what ‘good thinking’ means. Such a decision might offer clarity to interprofessional teaching and practice, or provide a foundation on which educational policy can be based. However, the critical thinking literature has long sought such a universal agreement and disagreements persist. Results of this study suggest a new approach, one that can account for multiple conceptions of critical thinking within and across health professions and practice contexts. The visual elicitation approach employed, asking participants to respond to the mind map, offered a unique perspective on the data that illuminated contradictions between conceptions held by individual participants, between participants, and between the conceptions themselves.

Such an approach offers a vehicle for thinking and talking about what kind of thinking is valued, both within and between professions. When conceptions of critical thinking are understood as flexible instead of stable, these acts of modification and contestation can be viewed as potential moments for critical self-reflection for individuals and for professional groups on the whole. Moreover, through their discussions of critical thinking, educators actively intervened to consider and assert what they value in their work.

These different conceptions might be complementary as often as they are incompatible. In fact, we would argue that ‘good thinking’ is inherently contentious (and should be) because it is such struggles over what ‘the good’ means in HPE that allow for challenges to the status quo. Advances at the heart of HPE and practice have been hard-won through deliberate reflection, discussion, action, and (often) conflict. For example, the ongoing movement toward relationship-oriented care has arguably occurred as a result of unexpected pushback regarding the limits of considering good healthcare as being entirely patient-centred. Thus, there is a need to bring unarticulated assumptions about important topics into the light so that the goals and values of educators and policy-makers can be openly discussed, even though they are unlikely to ever be fully resolved.

### Strengths and limitations

This study offered a broad sample of educators from four different professions, who practised in a range of disciplinary contexts. Given that the sampling approach taken sought breadth rather than depth, the results explore a range of conceptions of critical thinking across HPE, rather than allowing strong claims about any one profession or context. The sample also focussed on conceptions of critical thinking within health professions education at specific institutions in Edmonton, Alberta. A multi-institutional study might build on these results to elaborate the extent to which each health profession has a core shared conception of critical thinking that translates across institutional settings. We expect that there may be significant differences between settings, given that what is meant by critical thinking seems to be highly contextual, even from moment to moment. Mapping aspects of context that impact how individuals and groups think about critical thinking would tell us much more about the values on which these conceptions are based.

Subsequent studies might also explore the extent to which conceptions of critical thinking among those identifying as ‘educators’ are comparable to those identifying as primarily ‘clinicians’. Although the boundary is definitely blurry, these groups engage in different kinds of work and participate in different communities, which we suspect may result in differences in how they conceive of critical thinking.

## Conclusions

Rather than attempting to ‘solve’ the debate about what critical thinking should mean, this study maps the various conceptions of this term articulated by health professional educators. Educators took up biomedical, humanist, and social justice-oriented conceptions of critical thinking, and their conceptions often shifted from moment to moment or from context to context. The ‘mapping’ approach adopted to study this issue allowed for an appreciation of the ways in which educators actively modify and contest educational and professional values, even within their own thinking. Because critical thinking appears to be both value and context driven, arriving at a single right definition or taxonomy of critical thinking is unlikely to resolve deep tensions around what ‘good thinking’ in HPE means. Moreover, such an approach is unlikely to be productive. Such tensions produce challenges for shared understanding at the same time that they produce a productive space for discussion about core issues in HPE.

## Caption Electronic Supplementary Material

Appendix A: Interview Guide for Initial Interview

Appendix B: Interview Guide for Second Interview

Appendix C: Mind Map
