# Supplementary material for: Constructing critical thinking in health professional education
Source: Perspect Med Educ. 2018 Apr 4;7(3):156–65. doi: 10.1007/s40037-018-0415-z (PMC6002289; doi:10.1007/s40037-018-0415-z)
Supplement: Supplementary file 1 — Appendix A: Interview Guide for Initial Interview [file 40037_2018_415_MOESM1_ESM.docx]

## Appendix A: Interview Guide for Initial Interview

| Core Questions | Prompts |
| --- | --- |
| What is your teaching background? What are your beliefs about teaching? | - Can you tell me a bit about how and why you became and educator?   - How long have you been teaching? - Can you tell me a bit about what you teach?   - What does that look like? - What does a “typical day” of teaching look like?   - Any other teaching roles? - What do you hope, in a perfect world, students will learn   - From you?     - Why is that important?     - Short term? Long term?   - From their program more broadly? From other educators?   - What do you hope they will value? |
| What does critical thinking mean? | - Tell me what you think of when you hear the term *critical thinking* (CT) - Have you heard critical thinking talked about in other ways?   - Friends and family   - Colleagues from your discipline   - Colleagues from another discipline   - Within your institution?   - In the literature or policy documents (e.g. your code of ethics)   - How are these other ideas different than yours?   - Are there any contexts in which you think about critical thinking differently? - Why is critical thinking important?   - In your profession? In healthcare? In society broadly? |
| What does it look like? | - Can you tell me a bit about someone that you think is a real “critical thinker”   - What kind of skills or abilities do you think he/she is putting together in order to do that?   - What effect has that had?   - Do you have an example of their critical thinking? - How do you know if a student is thinking critically?   - Can you provide an example?   - How do you know if you’re *not* thinking critically? - You have also used the terms ___________. How are they related to CT? Are they the same thing? Different? - At the end of that, can you give me a definition of CT? |
| How did you come to this perspective? | - Why is critical thinking of interest to you?   - What clinical and personal experiences have reminded you of the importance of critical thinking? - How did you first understand CT as a learner? - What experiences as a learner have shaped how you think about CT?   - Any that changed how you think about critical thinking? - What teaching experiences have shaped how you think about CT?   - Any that changed how you think about critical thinking? - Have you had any experiences as a patient or family member that impact how you think about CT? |
| Discuss teaching artefacts | I asked you to bring in an example of a teaching artefact or assessment that you use to promote critical thinking in your students…   - Can you tell me a bit about this artefact? In what context(s) is it used? - Why did you choose it? - So if you gave a student ________, what do you hope would happen? - How would you know if it’s not working? Why wouldn’t it work? - What would you need to do to support it? - What other objects did you consider bringing today? Why did you decide not to use them? - Can you assess critical thinking? If so, how?   - What do you mean by assessment?   - What kind of informal feedback on critical thinking might you give? |
| Review | - At the beginning of the interview, you said CT means _________ to you. Would you change that now?   - What about this conversation has changed that? - Is there anything else that’s changed for you? New ideas? |
