# Supplementary material for: Constructing critical thinking in health professional education
Source: Perspect Med Educ. 2018 Apr 4;7(3):156–65. doi: 10.1007/s40037-018-0415-z (PMC6002289; doi:10.1007/s40037-018-0415-z)
Supplement: Supplementary file 2 — Appendix B: Interview Guide for Second Interview [file 40037_2018_415_MOESM2_ESM.docx]

## Appendix B: Interview Guide for Second Interview

Introductory statement

I am going to present you with a mind map that represents the terms and ideas about critical thinking (CT) that I heard from all participants in interview 1. You don’t have to agree with all of them – I expect most people will see themselves in some areas more than others. Critical thinking can mean different things in different contexts.

Start by looking over the map and finding one or two places that best match your idea of what critical thinking means. I’ll give you a few minutes to familiarize yourself with the map, then we’ll start talking through what you think about it. Please mark up the map – mark things that resonate for you and cross out things that don’t. Please ‘think-aloud’ so that I can get a sense of why you’re making the connections you are. I will ask follow up questions about why you’re going in directions you’re going.

| Core Questions | Prompts |
| --- | --- |
| What areas resonate most? | - Can you tell me how/why you chose to start there?   - Is there anything you would add to it? Or change about it? |
|  | - What other ideas resonate for you?   - How are they related to the concepts you started with? |
|  | - Are there any other ideas on here that relate to CT?   - How do they relate to your other ideas?   - Why didn’t you identify them initially? |
| Which ideas don’t fit for you? | - Are there ideas on the map that do not fit with your conception of CT?   - How are they different?   - Why do you think other people would see critical thinking this way? |
| How do the concepts on the map connect? | - Is there anything you would like to change about how the concepts are represented on this map?   - How would you draw this map differently?   - What works well for you about it? |
| Explore other areas | - Is there anything else that you would like to emphasize or remove from the map?   - We haven’t talked about X, what do you think about it?   - What made you decide to change/add/remove that? |
| How did you use the mind map? | - How did you feel about interacting with the map?   - How did you go about approaching the map?   - Did you identify familiar language right away or did it take a while?   - Did it look foreign? Overwhelming? |
| I have a couple of additional questions to clarify some of the ideas that came up in the first interview, not directly related to the mind map? | |
| How would you describe your family culture? | - What was valued within your family, growing up?   - How was did your family culture impact you?   - How does it relate to your beliefs about education/CT today? |
| How would you describe your profession? | - Positive aspects? Negatives? - How do you fit with your profession? - How does that “fit” create differences in terms of how you and others in your profession think? |
