# Supplementary material for: Constructing critical thinking in health professional education
Source: Perspect Med Educ. 2018 Apr 4;7(3):156–65. doi: 10.1007/s40037-018-0415-z (PMC6002289; doi:10.1007/s40037-018-0415-z)

## Appendix C: Mind Map

This visual depiction of interview one data takes the form of a “mind map,” generated using an open source web application. It was used to prompt discussion in interview two.


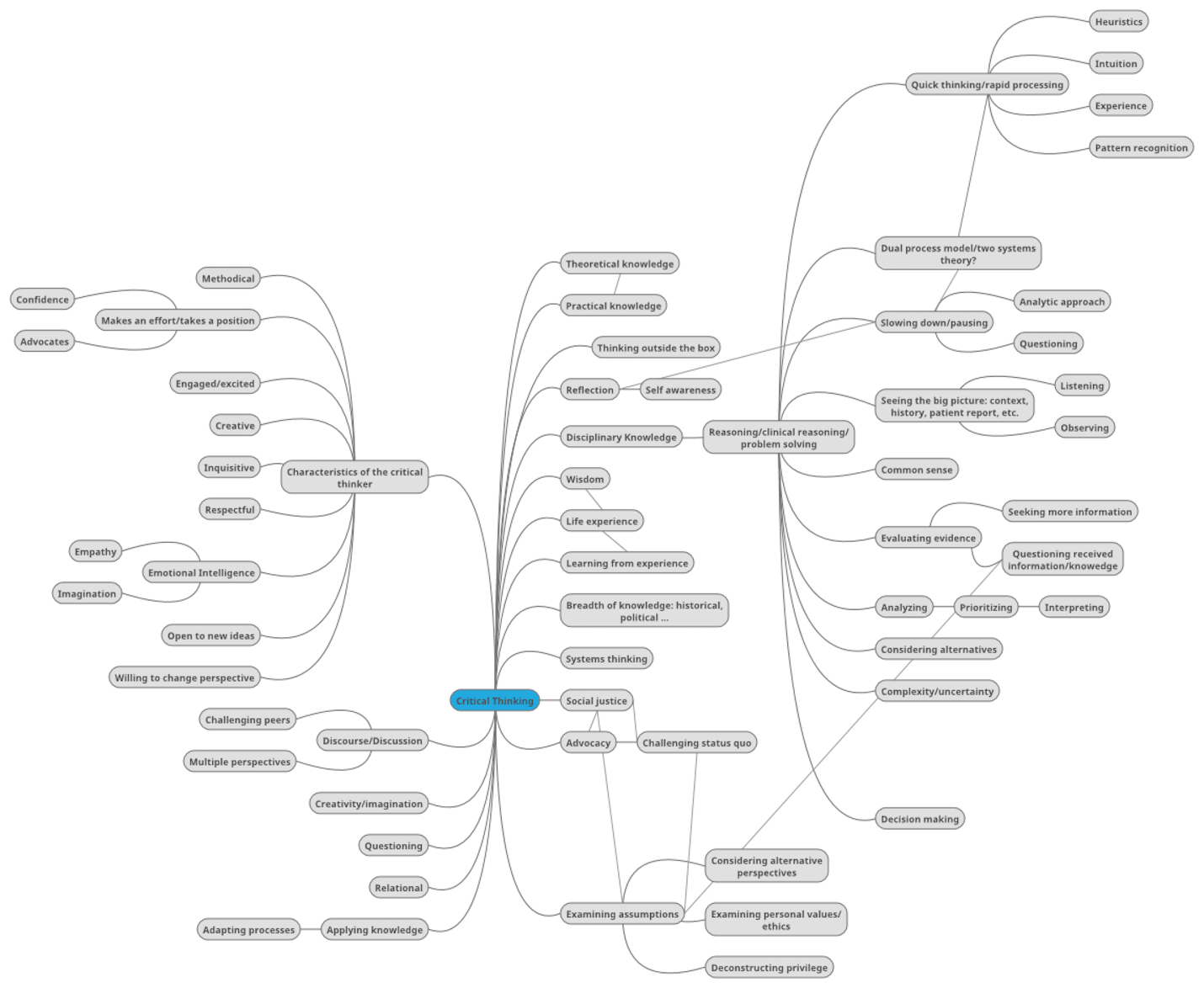

Supplement: Supplementary file 3 — Appendix C: Mind Map [file 40037_2018_415_MOESM3_ESM.docx]
